# The mind-body relationship in psychotherapy: grounded cognition as an explanatory framework

**DOI:** 10.3389/fpsyg.2014.00472

**Published:** 2014-05-20

**Authors:** Nuwan D. Leitan, Greg Murray

**Affiliations:** Department of Psychological Sciences and Statistics, Faculty of Health, Arts and Design, Swinburne University of Technology, HawthornVIC, Australia

**Keywords:** embodiment, embodied cognition, psychosocial treatments, psychotherapy, naturalism, phenomenology, mind–body, grounded cognition

## Abstract

As a discipline, psychology is defined by its location in the ambiguous space between mind and body, but theories underpinning *the application of psychology in psychotherapy* are largely silent on this fundamental metaphysical issue. This is a remarkable state of affairs, given that psychotherapy is typically a real-time meeting between two embodied agents, with the goal of facilitating behavior change in one party. The overarching aim of this paper is to problematize the mind–body relationship in psychotherapy in the service of encouraging advances in theory and practice. The paper briefly explores various psychotherapeutic approaches to help explicate relationships between mind and body from these perspectives. Themes arising from this analysis include a tendency toward dualism (separation of mind and body from the conceptualization of human functioning), exclusivism (elimination of either mind or body from the conceptualization of human functioning), or mind–body monism (conceptualization of mind and body as a single, holistic system). We conclude that the literature, as a whole, does not demonstrate consensus, regarding the relationship between mind and body in psychotherapy. We then introduce a contemporary, holistic, psychological conceptualization of the relationship between mind and body, and argue for its potential utility as an organizing framework for psychotherapeutic theory and practice. The holistic approach we explore, “grounded cognition,” arises from a long philosophical tradition, is influential in current cognitive science, and presents a coherent empirically testable framework integrating subjective and objective perspectives. Finally, we demonstrate how this “grounded cognition” perspective might lead to advances in the theory and practice of psychotherapy.

## INTRODUCTION

As a discipline, psychological science is “mounted above the philosophical gap between mind and body” (Tschacher and Haken, 2007, p. 1). The inherent challenges of this position are clearly seen in psychology’s primary application, psychotherapy (the use of psychological science to improve mental health and wellbeing). The theoretical foundation of psychopathology (the study of the nature and treatment of mental disorders) has been described as akin to that of biology’s before Darwin (Frances and Egger, 1999), and arguably, the elephant in the room is the lack of consensus, both implicit, and explicit, about the relationship between mind and body (Kendler, 2008). Whether expressed as human versus natural sciences, hermeneutic versus positivist methods, or understanding versus explanation, Cartesian or substance dualism (mind and body are two types of substance) is yet to be resolved in psychopathology and psychotherapy. The field is consequently characterized by polarized schools of thought, identifying it as an immature science in Kuhnian terms (Kuhn, 1962).

In the absence of a consensus position on the mind–body relationship, psychotherapists juggle tangible and intangible features of their clients without integrative models (Murray, 2011). It is noteworthy that international guidelines for psychology training programs rarely require a competency around this ontological issue, suggesting that the discipline may have relegated it to the “too hard” basket. Contemporary research across multiple disciplines, however, suggests that the case should be re-opened.

Recent research in philosophy (Clark, 1997; Lakoff and Johnson, 1999), cognitive science (Brooks, 1991; Chemero, 2009) and psychology itself (Barsalou, 1999; Glenberg and Robertson, 1999) advocates a fundamental reappraisal of the relationship between mind and body. The “embodied cognition” research program has many strands, but all commence with a rejection of the dualistic separation of body and mind (Shapiro, 2011). Here, we propose grounded cognition as an embodied, psychological framework which provides a holistic conceptualization of body and mind. It is our position that articulating the relationship between body and mind from a psychological perspective will provide a consensus position and an organizing framework for the mind–body relationship for psychotherapy research and practice. We contend that this will encourage practitioners to reflect on their assumptions about cognitions and how they conceptualize body and mind in treatment, leading to a better understanding of the tensions between psychotherapy theory and practice and the identification of gaps in existing therapies and consequently an expansion of the range of therapies offered to the patient.

The paper is structured in four sections. First, we briefly consider a range of approaches to psychotherapy through the lens of their apparent assumptions about mind–body. Themes arising from this analysis include a tendency toward an uncritical dualism (separation of mind and body from the conceptualization of human functioning), exclusivism (elimination of either mind or body from the conceptualization of human functioning), or mind–body monism (conceptualization of mind and body as a single, holistic system) and we conclude that the psychotherapy literature, as a whole, does not demonstrate consensus, regarding the relationship between mind and body. We propose that an organizing framework for the mind–body relationship, underpinned by a holistic conceptualization of the relationship, would benefit psychotherapy research and practice. Second, philosophical accounts which portray a holistic mind–body relationship from phenomenological and objective perspectives are outlined. Third, we propose that these perspectives are integrated, psychologically, by “grounded cognition,” constituting a comprehensively articulated, empirically informed, organizing framework for conceptualization of the mind–body relationship in psychotherapy. In the final section we consider how the application of psychological science in psychotherapy might advance through a thoroughgoing consideration of “grounded cognition.”

## MIND–BODY ASSUMPTIONS UNDERLYING CURRENT PSYCHOTHERAPIES

There is no agreed taxonomy of psychological therapies (e.g., Kahl et al., 2012; Tschacher et al., 2014), but to achieve an adequate coverage of existing approaches for the present purposes, we categorise psychotherapies into five fuzzy-bordered groups: psychoanalysis, behavioral therapies, cognitive therapies, mindfulness-based therapies, and body psychotherapies. Each of these has many branches and extensive literatures – thus, in this brief review we aim only to explore different ideas regarding the relationship between mind and body from within each approach, and across approaches, rather than attempting to assign particular conceptualizations of the mind–body relationship to particular approaches.

### PSYCHOANALYSIS

Although psychoanalytic theory and practice have fallen out of favor in contemporary psychological science, aspects of Freud’s thinking can still be discerned in current psychotherapy (Dowd, 2004). An important aspect of psychoanalytic theory is the “cognitive unconscious,” or the “unconscious mind.” In opposition to the popular enlightenment view at the time, Freud argued that behavior is driven by unconscious motivations and drives, rather than rational choice (Luborsky et al., 2008; Wolitzky, 2011). As discussed by Luborsky et al. (2008), central therapeutic strategies of psychoanalysis include free association (expressing any thoughts which come to mind during therapy), therapeutic listening and responding (examining the content and emotion of thought), and interpretation (drawing inferences about unconscious underpinnings of conscious experience).

However, the body also figures strongly in psychoanalytic theory. For Freud, structures of the mind (e.g., id, ego, superego) arise out of tensions between the organism’s bodily drives and societal structures (Muller and Tillman, 2007). This is reflected in the psychoanalytic conception of psychosomatic illness, which was the idea that emotions and unconscious desires caused bodily symptoms; for example Gregor Groddeck, a psychoanalyst who developed Freud’s ideas about psychosomatic illness proposed that a tumorous abdominal growth could result from a warded-off unconscious wish to be pregnant. Furthermore it has been suggested that the “ego,” in psychoanalysis, commences as an embodied entity, and emphasizes the continuity between animals and humans, suggesting a monist, or holistic mind–body conceptualization (Muller and Tillman, 2007).

### BEHAVIOR THERAPY

Traditional behavior therapy arose in an American setting in the early 1950s and saw a shift from the psychoanalytical ideas of studying the mind to the pragmatic, evidence-based study of behavior (Dowd, 2004). This shift was triggered by J. B. Watson’s criticism of subjectivity and mentalism as the subject matter of psychology and his advocacy of the objective study of behavior. This was followed by the advent of “modern learning theory,” which referred to the principles of classical and operant conditioning. These early ideas underlying traditional behavior therapy were exclusivist, rejecting the notion of mind and cognition, on the grounds that they are unobservable entities and therefore unfit for scientific study (Wilson, 2008; Zinbarg and Griffith, 2008).

However, later theories stemming from behaviorism developed a more complex account of the mind–body relationship. For example, Bandura (1977) spoke of a reciprocal determination between behavior and the environment, stating that “it is largely through their actions that people produce the environmental conditions that affect their behavior in a reciprocal fashion” (p. 345). Bandura also seemed to encourage conceptualization of the mind as a part of the same system as behavior and environment, for example, “…experiences generated by behavior also partly determine what individuals think, expect, and can do, which in turn affects their subsequent behavior” (p. 345).

This holistic conceptualization of the mind–body relationship is also apparent in popular behavior therapies for children with autism spectrum disorder, such as music therapy, Floortime, rhythm therapy, and reciprocal imitation training which are broadly underpinned by behavioral and functional developmental approaches (Greenspan and Wieder, 1999; Ingersoll and Schreibman, 2006; Overy, 2008; Vismara and Rogers, 2010; Srinivasan and Bhat, 2013). For example, reciprocal imitation training teaches children the spontaneous social use of imitation, which as targeted at attention, language and communication cognitions (Ingersoll and Schreibman, 2006) and Floortime utilizes child-led playful interactions, experiential problem-solving interactions and motor, sensory and spatial play, which is targeted at language and other cognitive skills (Greenspan and Weider, 1997).

### COGNITIVE THERAPY

With the advent of the cognitive revolution, pure behavioral therapies begun to fade out in favor of cognitive therapies, which followed the prominent model of human functioning at the time; computational theory (Hayes et al., 1999). Computational theory conceptualized the body as an “input-output device,” or the “hardware,” and the mind as the “central processor,” the “software,” or the “controller” (Shapiro, 2007). Due to their concurrent rise, articulation of the relationship between mind and body in cognitive therapy has been influenced by this computational perspective (Dowd, 2004).

Cognitive therapies are defined by their elevation of the cognitive system in the adjustment of information processing and initiation of positive change (Beck and Weishaar, 2008). This perspective is fundamental to a family of theories underpinning cognitive therapy, including those of Ellis (1962) and Beck (1967). Beck’s (1967) cognitive theory remains one of the most influential to this day, in particular his major contribution to cognitive therapy, the cognitive model (Triad) of depression. This model suggests that depression is underpinned by automatic, negative thoughts about the self, others and the world. Beck contends that these negative cognitions also activate negative motivational, behavioral, emotional, and physical symptoms (Beck and Weishaar, 2008). Thus, for Beck and his contemporaries, it is implied that the mind should be the primary target of psychotherapy.

One of Ellis’ major contributions to cognitive therapy was the A-B-C method used in his rational emotive behavior therapy (REBT). The A-B-C method challenged the assumption that when a consequence (C) follows and activating event (A), A causes C. Ellis posited a cognitive construct, beliefs (B), which he argued was the greatest determinate of (C). Thus, the idea was that (C) could be modified by (B), even if (A) remains stable (Dowd, 2004; Ellis, 2008). Ellis’ REBT explicitly considered the importance of content of the “mind” (i.e., thinking, feeling, wanting etc.), and of operations of the “body” (i.e., behavior). However, the relationship between mind and body was conceptualized in terms of cognitive modification to change behavior or behavior change to modify thought (Ellis, 2008). Thus, despite acknowledgment of both mind and body, REBT, akin to Beck’s cognitive therapy, implies a dualist conception of their relationship.

### MINDFULNESS-BASED PSYCHOTHERAPIES

Recently, there has been an influx of so-called “third wave” psychotherapies which have their roots in learning theory and are held together by their subordination of content-oriented cognitive interventions (Kahl et al., 2012). One of the key features of *some* of these psychotherapies (e.g., Acceptance and Commitment Therapy, Mindfulness-Based Cognitive Therapy etc.) is their focus on “mindfulness.” One of the features of mindfulness as applied in psychological therapies is to develop an awareness of the present experience by self-regulating attention to momentary sensations, thoughts, and feelings (Keng et al., 2011). Thus, in contrast to standard cognitive and behavioral therapies, one of the aims of mindfulness-based psychotherapies is to increase awareness of the body.

Awareness is contrasted with “thinking” during mindfulness exercises such as breathing meditation (Michalak et al., 2012). Awareness is not about cognition but more about feeling; and the body is seen as the reference point for awareness. Thus changes in cognitions (e.g., restricting rumination) following mindfulness practices are bought about by becoming more aware of the body, without referring to cognitive dominion (i.e., conscious thought) to bring about this awareness (Burg and Michalak, 2011). It is difficult to articulate the relationship between mind and body implied by mindfulness-based psychotherapies due to two reasons. First, awareness is not conceptualized as a cognitive feature, but may still be a feature of the “mind.” Second, the body is not conceptualized as a physical agent of change like behavior is assumed to influence cognition in cognitive behavior therapy (CBT); rather it is awareness of the body which is the agent of change in mindfulness-based therapies. These questions illustrate some of the issues which arise when dualistic thinking is reflected upon carefully.

### BODY PSYCHOTHERAPY

Body psychotherapy (BP) refers to a variety of schools (e.g., dance/movement therapy, analytical body psychotherapy, concentrative movement therapy etc.) which share the aim of enhancing self-awareness, modifying behavior, and facilitating insight-oriented psychological problem solving via a mode of action concerning perceptive/self-awareness, affective-cathartic, interactive, and/or movement oriented therapy (Röhricht, 2009). Although, there have been randomized controlled trials (RCTs) conducted for some schools of body psychotherapies, they are not empirically supported to the same extent that cognitive and behavioral therapies have been (Röhricht, 2009). In practice, BP primarily works on releasing and re-shaping somatic memories in order to release associated psychological constraints (Totton, 2003). The theoretical foundation for BP has been explained as the way “core beliefs are embodied, and that until we begin to experience the pain held in them directly through our bodies they will continue to run our lives” (Staunton, 2002, p. 4).

The practice of BP implies a very close relationship between body and mind, to the point that they are seemingly undifferentiated during therapy. BP has been described as being fundamentally underpinned by an explicit theory of mind–body functioning which assumes a functional unity between body and mind in which there is no separation or hierarchical relationship between the two (www.eabp.org).

### SUMMARY

This brief review exposes a lack of consensus, both implicit and explicit, regarding the mind–body relationship across psychotherapeutic approaches. Themes arising from this analysis include a tendency toward dualism (separation of mind and body from the conceptualization of human functioning), exclusivism (elimination of either mind or body from the conceptualization of human functioning), or mind–body monism (conceptualization of mind and body as a single, holistic system). It is our position that psychotherapeutic research and practice would benefit from an organizing framework for the mind–body relationship, which could be applied across all psychotherapies. Recent research in philosophy (Clark, 1997; Lakoff and Johnson, 1999), cognitive science (Brooks, 1991; Chemero, 2009) and psychology itself (Barsalou, 1999; Glenberg and Robertson, 1999) suggests that this framework should be underpinned by a holistic conceptualization of the mind–body relationship.

Embodied cognition offers a psychological framework underpinned by a holistic conceptualisation of the mind–body relationship. Some of the abovementioned psychotherapies which have *implied* a holistic mind–body perspective have already started to draw on embodied cognition and related ideas. For example, Totton (2009) has recently highlighted the utility of drawing on embodiment from a social perspective to enhance the practice of body psychotherapy, while Michalak et al. (2012) has described how embodied cognition could describe some of the processes involved in mindfulness. Before describing the psychological framework of embodied cognition, it is important to briefly examine its philosophical underpinnings which form the foundation for its conceptualisation of a holistic mind–body relationship, from both phenomenological and objective perspectives.

## HOLISTIC MIND–BODY PHILOSOPHIES

### MERLEAU-PONTY’S LIVED-BODY

Edmund Husserl developed the philosophical approach of phenomenology as a reaction to his concern that the assumptions of naturalistic, Western science about the nature of the mind, body, and world had caused it to miss fundamental questions about human nature (Marcum, 2004). He argued that primary consideration should be given to the subject’s experience in the world, before studying the mind, body, and world objectively (Marcum, 2004; Gallagher and Zahavi, 2007). Husserl’s argument was progressed by Merleau-Ponty, who proposed that this would both uncover the subjective element of knowledge, which was being overlooked by naturalistic sciences, and provide a stronger framework for its enquiries (Gallagher and Zahavi, 2007). Thus, phenomenology does not provide a mechanistic account of mind in the vein of naturalism, or psychological and biological accounts because it focuses on giving a proper description of humans’ experience in life, rather than attempting to forge an objective account of mind (Gallagher and Zahavi, 2007; Marshall, 2008).

Merleau-Ponty’s phenomenology argues for the prioritization of the subjective, lived-body in cognition and more specifically that cognitions cannot be understood without reference to the body which engages with the world (Merleau-Ponty, 1962, 1965; Marshall, 2008). Merleau-Ponty provides a comprehensive theory of the “lived-body,” or the “subject-body,” contrasting it to the “thing-body,” or the “object-body” (Merleau-Ponty, 1962, 1965; Marshall, 2008). The subject-body can be considered the body experienced from a first-person perspective which acts on the world, whereas the object-body can be considered the body as an object of the world experienced from a third-person perspective. Merleau-Ponty emphasizes the subject-body in cognition, implying that humans fundamentally are, and thus should be studied as embodied beings who form cognitions via interaction in the world with their bodies, rather than cognition as an activity of the “mind” which utilizes the object-body (Merleau-Ponty, 1962, 1965; Borrett et al., 2000; Matthews, 2004).

### DEWEY’S PRINCIPLE OF CONTINUITY

In contrast to Merleau-Ponty’s phenomenological approach, an alternative holistic account of the mind–body relationship starts from an objective position. American pragmatism offers an objective, philosophical account of a holistic mind and body in the form of naturalism (Johnson, 2006). As Horst (2002) explicates, there have been various definitions and strands of naturalism. The account we refer to in this section aligns with the Darwinian paradigm and, more specifically with physicalism, emergence, and supervenience (Harbecke, 2013; Montero, 2013; McLaughlin and Bennett, 2014).

This form of naturalism is committed to an account in which all things in the world, including body and mind are natural or *naturally emergent* (Horst, 2002; Aikin, 2006). In turn, it posits that all explanation should be causal and reducible to natural explanations and is consequently committed to the study of the person as an *object* and the natural evolution of all human functions (Aikin, 2006; Johnson, 2006). One account of naturalism, from this emergent, supervenient perspective is Dewey’s “principle of continuity” (Dewey, 1981, 1991).

The principle of continuity posits that there is no break in experience between the processes of perceiving, feeling, moving, and thinking; instead they are levels of organic functioning from which higher function emerges. It describes three levels of organization: the “physical” level of inanimate material processes; the “psycho-physical” level of living things which have needs, interests, and satisfactions; and the “mental” level of organisms which can perform higher level cognitions. The principle explains the progression from the physical level to the level of the mind without introducing new ontological entities, structures, or forces. Dewey argues that new organization is the reason that organisms with minds can do things which psycho-physical entities cannot do, and why psycho-physical entities can do things which physical entities cannot do. Thus, according to Dewey, what we refer to as “mind” is a complex *new organization* of what we refer to as “body,” but they are in essence the same entity. According to the principle of continuity, what is termed “mind” and “body” are simply ways to identify aspects of the organism–environment interaction which have arisen from an organic process (Dewey, 1981, 1991; Johnson, 2006, 2007).

### PHENOMENOLOGY AND NATURALISM AS COMPLEMENTARY APPROACHES

Phenomenology is committed to describing subjective experience, which is where meaning putatively arises for humans, while naturalism as characterized here provides an objective explanation of how meaning arises ontogenetically, organically and biologically, independent of the personal experience of the individual (Gallagher and Zahavi, 2007; Marshall, 2008). As Aikin (2006, p. 326) puts it “Lovers may love, and pains may pain, but the naturalistic perspective can attend only to the lovers, not their love; to the pains, but not their feelings of pain.” Similarly, the phenomenological perspective can attend only to the love, not the lovers and to the feelings of pain rather than the pains. Thus, phenomenologists can provide to naturalists, psychologists and neuroscientists a more precise model of the phenomenon which they attempt to explain than they would if they were to start only with an “objective” scientific theory of cognition (Gallagher and Zahavi, 2007). Thus, phenomenology and naturalism are contrasting, but complementary approaches (Aikin, 2006; Zahavi, 2010).

Accordingly, the different directions from which Merleau-Ponty’s phenomenology and Dewey’s principle of continuity approach the question of the relationship between mind and body are complementary, providing ultimately a more comprehensive, pluralistic understanding of the holistic mind–body relationship. Merleau-Ponty’s phenomenological account can inform Dewey’s objective account of how a person experiences the holistic mind–body described in his theory.

Thus, a philosophical integration of these perspectives may be possible (Zahavi, 2010), but our aim here is to provide a framework for psychotherapeutic research and practice. Therefore, it is necessary to provide a psychological account which integrates subjective and objective perspectives of a holistic mind–body relationship. We propose that grounded cognition provides such a framework.

## GROUNDED COGNITION AS A PSYCHOLOGICAL FRAMEWORK REFLECTING A HOLISTIC MIND–BODY RELATIONSHIP

Embodied cognition is a research program consisting of a number of accounts and topics, held together by the underlying assumption that the body functions as a *constituent* of the mind rather than a perceiver and actor serving the mind, thus being *directly,* and *subjectively* involved in cognition (Borrett et al., 2000; Shapiro, 2007). Different accounts of embodied cognition provide various models of this underlying assumption, so it is useful to focus on one to explore the holistic conceptualization of body and mind and how it aligns with the principles of Merleau-Ponty’s phenomenology and Dewey’s principle of continuity.

“Grounded cognition” reflects the underlying embodied cognition assumption by proposing that cognition is derived from, and dependent on, bodily interactions with the world which are represented in the brain (Barsalou, 2008). Grounded cognition has been comprehensively articulated and critiqued in the literature (Barsalou, 1999, 2008), has a strong empirical foundation (e.g., Schubert, 2005; Chandler and Schwarz, 2009; Jostmann et al., 2009; Natanzon and Ferguson, 2012 etc.) and most importantly, clearly explicates the holistic relationship of body and mind, aligning with both Merleau-Ponty’s phenomenology and Dewey’s principle of continuity as considered next.

Grounded cognition is underpinned by two major assumptions, namely that cognition is dependent on the body’s interaction with the world and that these interactions are represented in the brain (Barsalou, 2008). Grounded cognition’s first assumption is illustrated neatly by Shapiro (2011) in considering the concept of a morel mushroom for Sally, a mycologist, Charles, a provencal chef, and Lucy, a young child. Sally conceptualizes a morel as an epigenous ascocarp, Charles conceptualizes a morel as a delicacy to be sautéed with butter, and Lucy conceptualizes a morel as the yucky thing she has to eat before being allowed dessert. Thus, each according to their bodily experiences with morels forms different conceptualizations of it. However, these concepts are not determinate: for example, if Lucy grows up to become a mycologist, her concept of a morel would be more similar to Sally’s. Furthermore, it is important to note that there is nothing stopping Sally, Charles, and Lucy from having the same concept for a morel, it is simply their differing bodily interactions with the morel which has determined their conceptualizations. Finally, it can be assumed that they have the same visual conceptualization of a morel; they all know one when they see it. However, if Lucy were to have been born blind, she would never be able to obtain the same concept of a morel as Sally and Charles. Thus, grounded cognition aligns with Merleau-Ponty’s phenomenology by emphasizing the importance of subjective body-in-the-world experience for cognition (Johnson, 2006).

The second major assumption of grounded cognition is that the body’s relationship with the world is represented in the brain (Barsalou, 2008). Theories within grounded cognition differ on how these bodily interactions are represented in the brain, with some theories positing “image schemas” of bodily interactions in the world which are proposed to underpin abstract conceptual knowledge (Lakoff and Johnson, 1999). However, most grounded cognition theories propose “simulations,” which are neural reconstructions of experience using representations contained in modal systems of the brain (Glenberg, 1997; e.g., the sensorimotor system; Barsalou, 1999; Gallese and Lakoff, 2005). Thus grounded cognition is also consistent with Dewey’s principle of continuity in that from an objective, neuroscientific perspective, cognitions are emergent from, and inextricably intertwined with the body.

In sum, grounded cognition implies that cognition is emergent from and inextricably tied to the subjective, lived, experience of the body-in-the-world. Thus, “mind” and “body” only function as labels attached to properties of human functioning which we *perceive* as originating either mentally or physically. Conceiving of the relationship between body and mind from this holistic, psychological perspective can be expected to have a number of important implications for psychotherapy theory and practice.

## IMPLICATIONS FOR PSYCHOTHERAPY THEORY AND PRACTICE

First, a holistic conceptualization of the mind–body relationship leads to a better understanding of the tensions between psychotherapy theory and practice. When the mind–body relationship is conceptualized from a dualist or exclusivist perspective, a tension is created between the phenomenological needs of the patient who is present mind *and* body and the emphasis on either mind *or* body according to the theoretical assumptions of the psychotherapy practiced by the therapist. One example of this is the de-emphasis of the body during the practice of psychotherapies whose underlying theory disembodies the mind. During such therapies (e.g., cognitive therapy), touch is purposefully excluded from therapeutic practice since the mind is conceptualized as the agent of change, even though therapeutic practice could possibly be enhanced by touch (Feltham, 2008).

Second, a psychologically articulated, holistic framework for the mind–body relationship encourages *theoretical* reflection about this relationship by challenging dualist and exclusivist assumptions inherent in some psychotherapies. In turn, this helps to clarify some of the points of difference between the psychotherapies described above. Numerous psychotherapies discussed in “Mind–Body Assumptions Underlying Current Psychotherapies,” have similar theoretical background and similar therapeutic practices. An example of this is traditional behavioral therapy and body psychotherapy. Both emphasize the body and conceptualize it as the agent of change and as a consequence, both prioritize the body in therapy. One of the primary differences between the two can be ascertained by reflecting on the mind–body relationship. Traditional behavior therapy is very much exclusivist, dismissing the mind and cognition and emphasizing the body and behavior, both methodologically and theoretically. Contrastingly, body psychotherapy recognizes cognitions whilst treating them via the body, thus implying a holistic conceptualization of mind and body.

Third, a holistic conceptualization of the mind–body relationship has the potential to further de-stigmatize mental illness (Thomas, 2013; Ungar and Knaak, 2013a,b). Ungar and Knaak (2013a) suggest that dismissive and blaming attitudes toward mental health issues can be attributed to the absence of an organic explanation for most mental health issues. Thomas (2013) suggests that promoting mental illness to non-psychiatric health professionals as an interaction between cognitive, behavioral, emotional, biological, and environmental factors would reduce dualistic thinking around mental health issues and help with de-stigmatization in these settings. The psychologically articulated, holistic conceptualization of the mind–body relationship presented here elaborates on Thomas’ idea by conceptualizing cognitive, behavioral, emotional, biological, and environmental factors as part of the same functional system, implying that “organic” causes are inseparable from “mental” causes. Thus, we propose that the holistic conceptualization of the mind–body relationship presented here will further help with de-stigmatization of mental illness in non-psychiatric settings.

Fourth, the clearly articulated, explicit position of a holistic mind–body portrayed by grounded cognition encourages a more reflective approach to the issue *in practice*. Theories underlying most current psychotherapies do not explicitly state their position regarding the relationship between mind and body. Consequently, practitioners unreflectively adopt the assumptions inherent in the psychotherapies they utilize. The clear articulation of a holistic mind–body from both phenomenological and objective perspectives may assist practitioners to reflect on this relationship. For example, from a grounded cognition perspective “mind” and “body” are only labels attached to properties of human functioning which we *perceive* as originating either mentally or physically. The issue for psychotherapy practice is that in using these labels with patients, they automatically divide psychopathologies into arbitrary categories and thus portray dualist or exclusivist agendas. This then restricts the patient’s conceptualization of what the psychopathology is and how to manage it. A grounded cognition perspective would encourage a broader language around psychopathologies as disorders of the “system,” whether the symptoms are perceived as mental or physical. This will encourage the patient to focus on the holistic nature of their symptoms during treatment, as opposed to the idea that some treatments are behavioral/bodily and others are mind/cognitive. This is but one example of changes which may come of reflecting on the mind–body relationship in practice.

Finally, a new perspective on the mind–body relationship will guide the identification of gaps in existing therapies and consequently promote an expansion of the range of therapies offered to the patient. For example, grounded cognition implies that one way to change cognitions is through the subjective, lived, bodily experience of the individual. Encouraging practitioners to reflect on a holistic mind–body approach may result in a wider range of therapies they can offer their patients stemming from this idea. Further development of these ideas may also result in the creation of new and innovative therapeutic methods to augment those already in existence.

## CONCLUSION

Psychological science sits awkwardly between mind and body, and its application in psychotherapy inherits this awkwardness in a lack of clarity about how therapists should conceptualize their patients. By reviewing how mind and body are traditionally understood in major psychotherapies, we have attempted to underscore some of the tensions in this area. By introducing and outlining grounded cognition as a holistic psychological approach consistent with both radically subjectivist (Merleau-Ponty) and objectivist (Dewey) philosophical approaches, we hope to have proposed a new way forward for theorists and practitioners of psychotherapy. This new way forward throws light on the relationship between existing psychotherapies, the relationship between theory and practice, and highlights opportunities for new approaches to psychotherapy.

## Conflict of Interest Statement

The authors declare that the research was conducted in the absence of any commercial or financial relationships that could be construed as a potential conflict of interest.
